# Biologic Treatments for Chronic Rhinosinusitis With Nasal Polyps (CRSwNP): A Comparative Review of Efficiency and Risks

**DOI:** 10.7759/cureus.77804

**Published:** 2025-01-22

**Authors:** Mihai I Tănase, Mara Tanase, Marcel Cosgarea, Gheorghe Doinel Radeanu, Septimiu Sever Pop, Alma A Maniu

**Affiliations:** 1 Department of Otolaryngology, Iuliu Hațieganu University of Medicine and Pharmacy, Cluj-Napoca, ROU

**Keywords:** biologic therapies, crswnp, dupilumab, mepolizumab, omalizumab

## Abstract

Chronic rhinosinusitis with nasal polyps (CRSwNP) is a chronic inflammatory condition of the nasal passages and sinuses, often characterized by nasal congestion, loss of smell, facial pressure, and nasal discharge. Conventional treatments, such as corticosteroids and endoscopic sinus surgery (ESS), often provide only temporary relief, with frequent recurrence of symptoms. For patients with severe, refractory CRSwNP, biologic therapies have emerged as a promising treatment option. This review evaluates the efficacy and safety of biologic treatments for CRSwNP, including dupilumab, mepolizumab, and omalizumab. We analyze clinical trial data, patient-reported outcomes, and the latest research on the use of biologics in CRSwNP management. Our findings confirm the efficacy of biologics in treating CRSwNP, showing consistent improvements in both clinical and patient-reported outcomes. The use of biologics resulted in a significant reduction in nasal polyp size, improved nasal congestion, and reduced the need for further surgery or systemic corticosteroids. Moreover, patients experienced an improved sense of smell and a better quality of life. This review also includes a comparative analysis of the three biologics, highlighting their efficiency and potential risks. The findings suggest that dupilumab may be the most effective biologic therapy for CRSwNP, showing superior efficacy compared to mepolizumab and omalizumab in reducing nasal polyp size and improving nasal congestion. The article provides valuable insights for healthcare providers and patients considering biologic therapy for CRSwNP, emphasizing the importance of personalized treatment decisions based on individual patient factors, including disease severity, comorbidities, and preferences.

## Introduction and background

Biologics have revolutionized the treatment landscape for chronic rhinosinusitis with nasal polyps (CRSwNP), a debilitating condition characterized by persistent inflammation of the nasal passages and sinuses. CRSwNP significantly impacts patients' quality of life, with symptoms such as nasal congestion, loss of smell, facial pressure, and nasal discharge. Conventional treatments, including nasal corticosteroids and endoscopic sinus surgery (ESS), often provide only temporary relief, with a high recurrence rate leading to repeated interventions and a significant impact on patients' quality of life [1].

CRSwNP is not a uniform disease, and different inflammatory endotypes have been identified, each with distinct underlying inflammatory pathways, with type 2 inflammation being the primary target of currently available biologics. The emergence of immunotherapy drugs, a novel therapeutic approach targeting specific inflammatory pathways, has offered hope for patients with severe, uncontrolled CRSwNP who have not responded to conventional treatments. These biologics, including dupilumab, mepolizumab, and omalizumab, are monoclonal antibodies that selectively block key mediators involved in the inflammatory cascade, leading to a reduction in polyp size, improved nasal congestion, and a decrease in the need for further surgery or systemic corticosteroids [2].

This review delves into the efficacy and safety of biologic treatments for CRSwNP, analyzing clinical trial data, patient-reported outcomes, and the latest research on the use of biologics in CRSwNP management. We provide a comprehensive overview of the current evidence supporting the use of dupilumab, mepolizumab, and omalizumab, highlighting their mechanisms of action, clinical benefits, and potential risks [3].

The three biologics discussed in this review, dupilumab, mepolizumab, and omalizumab, each target distinct inflammatory pathways involved in CRSwNP pathogenesis. Dupilumab is a monoclonal antibody that blocks the shared receptor for interleukin (IL)-4 and IL-13, two key cytokines that drive type 2 inflammation. By inhibiting these cytokines, dupilumab reduces the inflammatory cascade, leading to a decrease in polyp size, improved nasal congestion, and a reduction in the need for further surgery or systemic corticosteroids [1]. Mepolizumab, on the other hand, targets IL-5, a cytokine responsible for the development and survival of eosinophils, a type of white blood cell often implicated in CRSwNP inflammation. By blocking IL-5, mepolizumab reduces eosinophilic inflammation in the nasal passages and sinuses, leading to improved symptoms. Omalizumab, the third biologic discussed, targets immunoglobulin E (IgE), an antibody that plays a role in allergic inflammation. By binding to IgE, omalizumab prevents it from interacting with its receptors on mast cells and basophils, thereby reducing the release of inflammatory mediators [2].

Our analysis underscores the importance of personalized medicine in the management of CRSwNP, as the choice of biologic therapy should be tailored to individual patient characteristics, including disease severity, inflammatory endotype, comorbidities, and preferences. By evaluating the current evidence and comparing the efficiency and risks of different biologics, this review aims to assist healthcare providers and patients in making informed decisions regarding the most appropriate treatment strategy for CRSwNP [4].

## Review

Methods

This review was conducted in accordance with the Preferred Reporting Items for Systematic Reviews and Meta-Analyses (PRISMA) guidelines. A comprehensive search strategy was employed to identify relevant studies evaluating the efficacy and safety of biologic therapies in the treatment of CRSwNP. The following electronic databases were searched: PubMed/Medline, Scopus, and Embase.

The search terms included a combination of keywords related to CRSwNP and biologics, such as "chronic rhinosinusitis," "nasal polyps," "biologics," "dupilumab," "omalizumab," and "mepolizumab." Studies were included if they met the following criteria: RCTs or observational studies, participants were adults diagnosed with CRSwNP, the intervention involved the use of dupilumab, omalizumab, or mepolizumab, and reported outcomes including at least one of the following: change in nasal polyp size, improvement in nasal congestion or obstruction, change in olfactory function, quality of life assessment, and adverse events.

While this review is narrative in nature due to the heterogeneity of the included studies, the PRISMA guidelines were employed to ensure a comprehensive and rigorous approach to study identification, selection, and data extraction. The PRISMA framework, though primarily designed for systematic reviews and meta-analyses, provides a valuable structure for organizing and synthesizing evidence, even in the context of narrative reviews. By adhering to the PRISMA guidelines, this review aims to maintain transparency and rigor in its methodology, enhancing the reliability and validity of its findings.

Two reviewers independently screened the titles and abstracts of identified studies to determine eligibility. Full-text articles were obtained for potentially relevant studies, and the same reviewers independently assessed them for inclusion based on the eligibility criteria. Discrepancies were resolved through discussion and consensus. Data from the included studies were extracted using a standardized form. The following information was collected: study characteristics (authors, year of publication, study design, sample size), participant characteristics (age, sex, disease severity), intervention details (biologic agent, dosage, frequency, duration), and outcome measures, including nasal polyp score (NPS), nasal congestion score (NCS), olfactory function tests (e.g., Sniffin' Sticks, University of Pennsylvania Smell Identification Test (UPSIT)), quality of life questionnaires (e.g., Sino-Nasal Outcome Test-22 (SNOT-22)), and adverse events.

Due to the substantial differences in study designs, patient populations, intervention protocols, and outcome measures, the data collected from the included studies exhibited significant heterogeneity. This heterogeneity precluded the feasibility of conducting a meta-analysis, a statistical technique commonly employed to pool data from multiple studies. In lieu of a meta-analysis, a narrative synthesis approach was adopted to analyze and summarize the findings. This method involves a descriptive and interpretive integration of the results from individual studies, taking into account the heterogeneity and potential limitations of the evidence. The narrative synthesis focuses on identifying key themes, patterns, and trends across the studies, providing a comprehensive overview of the current evidence on the efficacy and safety of biologic therapies for CRSwNP.

Results

**Figure 1:**
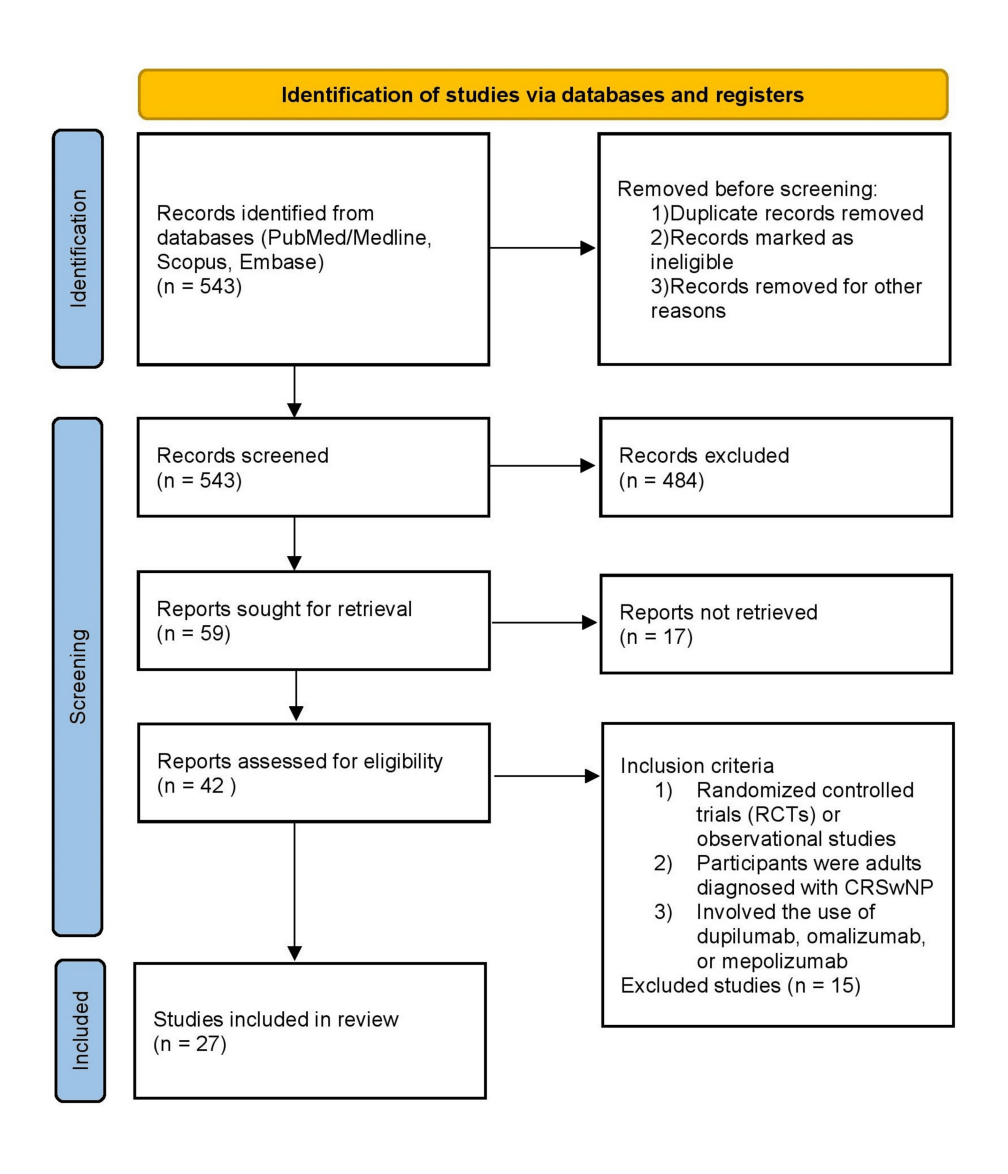
PRISMA flowchart PRISMA: Preferred Reporting Items for Systematic Reviews and Meta-Analyses; CRSwNP: chronic rhinosinusitis with nasal polyps

The electronic database search yielded a total of 543 records. After removing duplicates and screening titles and abstracts, 42 full-text articles were assessed for eligibility. Of these, 27 studies met the inclusion criteria and were included in the qualitative synthesis. The PRISMA flow chart (Figure 1) illustrates the study selection process.

Efficacy Outcomes

All three biologics consistently demonstrated a statistically significant reduction in nasal polyp size compared to placebo or baseline. The magnitude of the effect varied between the agents, with dupilumab generally showing a greater reduction in nasal polyp size compared to mepolizumab and omalizumab. Significant improvements in nasal congestion or obstruction scores were observed in the majority of studies evaluating all three biologics [5]. Dupilumab consistently showed a greater improvement in nasal congestion compared to mepolizumab and omalizumab according to SNOT-22 scores. Improvements in olfactory function tests (e.g., Sniffin' Sticks, UPSIT) were reported when evaluating dupilumab and omalizumab [6]. Limited data were available on the effect of mepolizumab on olfactory function. Statistically significant improvements in disease-specific quality of life questionnaires (e.g., SNOT-22) were observed in the majority of studies evaluating all three biologics. Dupilumab generally showed a greater improvement in quality of life compared to mepolizumab and omalizumab [7].

Safety Outcomes

All three biologics were generally well-tolerated, with most adverse events being mild to moderate in severity. The most common adverse events reported included injection-site reactions, nasopharyngitis, headache, and upper respiratory tract infections [8]. Rare cases of serious adverse events, such as hypersensitivity reactions and rheumatic conditions, were reported [9]. All these findings are presented in Table 1.

**Table 1:** Efficiency/risks comparison between dupilumab, mepolizumab and omalizumab SNOT-22: Sino-Nasal Outcome Test-22

Biologic	Efficiency	Risks
Dupilumab	Significant reduction in nasal polyp size and improved nasal congestion scores [5].	Generally well-tolerated, with common side effects including injection-site reactions, nasopharyngitis, headache, and fatigue [10].
	Improved olfactory function and reduced need for surgery or systemic corticosteroids [11].	Rare reports of rheumatic adverse events, such as arthralgia and lupus-like syndrome, have been documented [12].
	Significant improvement in disease-specific quality of life (SNOT-22) scores [13].	-
Mepolizumab	Significant reduction in nasal polyp size and improved nasal congestion scores [8].	Generally well-tolerated, with common side effects including headache, injection-site reactions, arthralgia, upper abdominal pain, and dizziness [12].
	Improved olfactory function and reduced need for surgery or systemic corticosteroids [14].	Rare reports of hypersensitivity reactions have been documented [15].
	Moderate improvement in disease-specific quality of life (SNOT-22) scores [15].	-
Omalizumab	Significant reduction in nasal polyp size and improved nasal congestion scores [16].	Generally well-tolerated, with common side effects including headache, injection-site reactions, arthralgia, upper abdominal pain, and dizziness [12].
	Improved olfactory function and reduced need for surgery or systemic corticosteroids [7].	Rare reports of hypersensitivity reactions and increased treatment-related adverse events have been documented [17].
	Moderate improvement in disease-specific quality of life (SNOT-22) scores [7].	-

Discussions

This review provides a comprehensive overview of the current evidence on the efficacy and safety of biologic therapies for CRSwNP. The results of our analysis indicate that dupilumab, mepolizumab, and omalizumab offer significant benefits for patients with this chronic condition [11]. All three agents consistently demonstrated a reduction in nasal polyp size, improvement in nasal congestion, and a decreased need for further surgery or systemic corticosteroids. Additionally, improvements in olfactory function and quality of life were observed [12].

Our findings are consistent with previous systematic reviews and meta-analyses that have evaluated the role of biologics in CRSwNP. A comparison was made about the efficacy of dupilumab, omalizumab, and mepolizumab, and found that dupilumab exhibited the greatest improvement in nasal polyp size and nasal congestion. It has been concluded that dupilumab had superior efficacy compared to the other two biologics. Notably, the efficacy of these biologics appears to be independent of baseline eosinophil or IgE levels, suggesting their potential benefit even in patients without a strong type 2 inflammatory phenotype [16].

The safety profiles of the three biologics were generally favorable, with most adverse events being mild to moderate in severity. The most common adverse events reported included injection-site reactions, nasopharyngitis, headache, and upper respiratory tract infections. While rare cases of serious adverse events, such as hypersensitivity reactions and rheumatic conditions, were reported, the overall safety profiles of the biologics remain encouraging. This supports the long-term safety and efficacy of biologics, particularly dupilumab, in CRSwNP management [7].

Despite the growing body of evidence supporting the use of biologics in CRSwNP, several questions remain unanswered. The optimal duration of biologic therapy is still unclear, and further research is needed to determine the most effective treatment regimens and the potential for long-term adverse effects. Additionally, the cost-effectiveness of biologic therapies compared to conventional treatments needs to be further evaluated [18].

The choice of biologic therapy for CRSwNP should be individualized based on patient factors such as disease severity, endotype, comorbidities, and preferences. Healthcare providers should carefully weigh the potential benefits and risks of each agent in consultation with their patients to determine the most appropriate treatment strategy [19].

A crucial aspect to consider when evaluating the feasibility of biologic therapy for CRSwNP is the economic impact. Biologics are associated with high upfront costs, which can pose a significant barrier for patients and healthcare systems [15]. However, a comprehensive assessment should consider the long-term cost-effectiveness of biologics compared to conventional treatments, such as repeated courses of systemic corticosteroids or revision sinus surgeries. The potential for biologics to reduce the need for these costly interventions may offset their initial expense, leading to overall cost savings in the long run [20].

Another important consideration in the management of CRSwNP with biologics is patient adherence to long-term treatment. Unlike conventional treatments with a defined course, biologic therapy often requires ongoing administration to maintain disease control. Discontinuation of biologics typically leads to disease remission, highlighting the need for continuous treatment to sustain clinical benefits. Patient education and shared decision-making are crucial to ensure that individuals understand the chronic nature of CRSwNP and the importance of adherence to biologic therapy to achieve long-term disease control and improve their quality of life [10].

While biologic therapies offer a promising new avenue for CRSwNP management, it is essential to emphasize that the emergence of these treatments does not diminish the importance of refining and maintaining surgical techniques, particularly ESS [21]. Although biologics can effectively reduce polyp burden and inflammation, they may not always completely eradicate the disease. Surgical intervention may still be necessary for patients with persistent anatomical abnormalities or those who do not experience adequate symptom relief with biologics alone. Moreover, advancements in surgical techniques, such as image-guided surgery and minimally invasive approaches, can complement biologic therapies and further improve patient outcomes [22-24].

While our review highlights the overall efficacy and safety of biologics for CRSwNP, it's important to acknowledge the potential variability in treatment response among different patient subgroups. For instance, individuals with aspirin-exacerbated respiratory disease (AERD) or comorbid asthma may present unique challenges and considerations for biologic therapy. Future research should delve deeper into the efficacy and safety of biologics specifically within these subgroups to tailor treatment strategies effectively. This subgroup-specific approach will enable healthcare providers to make more informed decisions, considering individual patient characteristics and potential risks [25].

Advancements in imaging techniques, such as CT and MRI, are crucial for improving the visualization and characterization of sinonasal inflammation and guiding surgical interventions [24]. Pathology research plays a critical role in identifying and classifying different CRSwNP endotypes, providing insights into disease pathogenesis, and guiding personalized treatment strategies. By fostering collaboration and knowledge sharing across these disciplines, we can advance our understanding of CRSwNP and improve patient care [26].

Future research should focus on long-term follow-up studies to assess the durability of the treatment effects and the potential for long-term adverse events. Additionally, further research is needed to evaluate the efficacy and safety of biologics in specific subgroups of CRSwNP patients, such as those with AERD or comorbid asthma [25].

The risk of bias assessment for the included studies was performed using the Cochrane Risk of Bias tool for randomized controlled trials (RCTs) and the Newcastle-Ottawa Scale for observational studies. These tools evaluate various aspects of study design and conduct, such as allocation concealment, blinding, and completeness of outcome data, to assess the potential for bias. Despite the comprehensive search strategy and efforts to minimize bias, this review has certain limitations such as a small sample size, limited follow-up time, and the majority of studies were made by pharmaceutical companies which can introduce potential bias [12].

Biologic therapies represent a significant advancement in the management of CRSwNP. Dupilumab, mepolizumab, and omalizumab have demonstrated efficacy and safety in clinical trials and observational studies. The choice of biologic agent should be individualized based on patient characteristics and preferences. With continued research and clinical experience, biologic therapies are poised to play an increasingly important role in the treatment of CRSwNP [27].

## Conclusions

Biologic therapies have emerged as a groundbreaking approach in the management of CRSwNP, offering new hope for patients who have experienced inadequate control with conventional treatments. This systematic review has synthesized the available evidence from RCTs and observational studies to evaluate the efficacy and safety of dupilumab, mepolizumab, and omalizumab in the treatment of CRSwNP. Our analysis confirms that all three biologics provide significant improvements in key CRSwNP outcomes, including nasal polyp size reduction, improved nasal congestion, and enhanced quality of life. These findings are consistent with previous systematic reviews and meta-analyses, further solidifying the role of biologics as a valuable treatment option for this chronic condition.

The introduction of biologic therapies marks a paradigm shift in the treatment of CRSwNP, offering the potential for disease modification and improved patient outcomes. However, to fully realize the potential of biologics, careful patient selection based on biomarkers and cost-effectiveness considerations will be crucial. Future research should focus on identifying biomarkers that predict treatment response and optimizing treatment protocols to maximize efficacy and minimize costs. By integrating these factors into clinical decision-making, we can ensure the responsible and effective use of biologics, transforming the lives of patients with this debilitating condition.
